# Cortico-cerebellar audio-motor regions coordinate self and other in musical joint action

**DOI:** 10.1093/cercor/bhac243

**Published:** 2022-06-30

**Authors:** Natalie Kohler, Giacomo Novembre, Katarzyna Gugnowska, Peter E Keller, Arno Villringer, Daniela Sammler

**Affiliations:** Department of Neurology, Max Planck Institute for Human Cognitive and Brain Sciences, Stephanstr. 1a, 04103, Leipzig, Germany; Research Group Neurocognition of Music and Language, Max Planck Institute for Empirical Aesthetics, Grüneburgweg 14, 60322 Frankfurt am Main, Germany; Neuroscience of Perception and Action Laboratory, Italian Institute of Technology, Viale Regina Elena 291, 00161 Rome, Italy; Department of Neurology, Max Planck Institute for Human Cognitive and Brain Sciences, Stephanstr. 1a, 04103, Leipzig, Germany; Research Group Neurocognition of Music and Language, Max Planck Institute for Empirical Aesthetics, Grüneburgweg 14, 60322 Frankfurt am Main, Germany; Center for Music in the Brain, Department of Clinical Medicine, Aarhus University, Universitetsbyen 3, 8000 Aarhus C, Denmark; The MARCS Institute for Brain, Behaviour and Development, Western Sydney University, Locked Bag 1797, Penrith NSW 2751, Australia; Department of Neurology, Max Planck Institute for Human Cognitive and Brain Sciences, Stephanstr. 1a, 04103, Leipzig, Germany; Research Group Neurocognition of Music and Language, Max Planck Institute for Empirical Aesthetics, Grüneburgweg 14, 60322 Frankfurt am Main, Germany; Department of Neuropsychology, Max Planck Institute for Human Cognitive and Brain Sciences, Stephanstr. 1a, 04103 Leipzig, Germany

**Keywords:** interpersonal synchronization, motor simulation, self-other integration and segregation, cerebellum, fMRI

## Abstract

Joint music performance requires flexible sensorimotor coordination between self and other. Cognitive and sensory parameters of joint action—such as shared knowledge or temporal (a)synchrony—influence this coordination by shifting the balance between self-other segregation and integration. To investigate the neural bases of these parameters and their interaction during joint action, we asked pianists to play on an MR-compatible piano, in duet with a partner outside of the scanner room. Motor knowledge of the partner’s musical part and the temporal compatibility of the partner’s action feedback were manipulated. First, we found stronger activity and functional connectivity within cortico-cerebellar audio-motor networks when pianists had practiced their partner’s part before. This indicates that they simulated and anticipated the auditory feedback of the partner by virtue of an internal model. Second, we observed stronger cerebellar activity and reduced behavioral adaptation when pianists encountered subtle asynchronies between these model-based anticipations and the perceived sensory outcome of (familiar) partner actions, indicating a shift towards self-other segregation. These combined findings demonstrate that cortico-cerebellar audio-motor networks link motor knowledge and other-produced sounds depending on cognitive and sensory factors of the joint performance, and play a crucial role in balancing self-other integration and segregation.

## Introduction

Many social interactions, from dyads clinking glasses to orchestras performing symphonies, require precisely timed group-level coordination. How well partners coordinate in time hinges on their ability to attend to, and estimate the timing of others’ actions, and to flexibly adapt their own actions accordingly (Keller et al. 2014). How this complex sensorimotor interplay between self and other is neurocognitively orchestrated is largely unknown. The present study applied functional magnetic resonance imaging (fMRI) in duetting pianists to fill this gap.

Joint action requires the pursual of 2 goals simultaneously—at the individual and the group level (Keller and Repp 2008; Knoblich et al. 2011; Vesper et al. 2017; Heggli et al. 2019a; Liebermann-Jordanidis et al. 2021). For example, ensemble musicians are required to plan and perform their own actions as accurately as possible (individual level) while flexibly coordinating and synchronizing with the actions of their co-performers (group level). To precisely perform one’s own part, self-generated action plans and sensory feedback need to be constantly monitored and segregated from other-generated action feedback (Keller et al. 2016). To synchronize with co-performers, other-generated action feedback has to be constantly integrated into self-generated action plans by attending to the temporal relationship between self- and other-produced feedback, and by adapting one’s own action timing, if necessary (Keller 2001). Both these processes—self-other integration and segregation—are often well balanced during joint performance, as this reduces cognitive effort and frees attentional resources (Keller 2001; Koban et al. 2019). However, a number of factors have been identified that may shift this balance towards stronger integration or segregation. Amongst those are external sensory, internal cognitive, as well as social factors, such as the temporal compatibility of partners’ action feedback (sensory), own motor experience with a co-performer’s action (cognitive), or personality traits (social; Goebl and Palmer 2009; Fairhurst et al. 2014; Novembre et al. 2016, 2019; MacRitchie et al. 2018; Heggli et al. 2019b). The goal of our study was to investigate how external sensory (temporal asynchrony of partners’ action feedback) and internal cognitive factors (motor familiarity with the partner’s actions) modulate interpersonal coordination, self-other processing and their neural correlates during joint music performance.

Asynchrony between the sounds of co-performers’ actions, ranging from subtle interpersonal differences in expressive timing (Keller et al. 2007; Ragert et al. 2013) to complementary timings of partners in the joint production of complex rhythmic patterns (Hofmann et al. 2017), is one factor that has been closely linked to self-other integration and segregation during joint music performance. In interpersonal synchronization tasks, humans typically adapt their actions mutually to their partner’s sounds to maintain low levels of temporal asynchrony, indicating well balanced self-other integration and segregation (Konvalinka et al. 2010; Heggli et al. 2019b). Even in the case of subliminal tempo incongruencies, humans automatically adapt their movement timing to synchronize with external rhythmic sensory events (Repp 2005; Repp and Su 2013). When timing incongruencies increase, interpersonal adaptation has been shown to decrease, indicating a shift towards stronger self-other segregation (Fairhurst et al. 2013; Novembre et al. 2016; Koban et al. 2019). Contrarily, when asynchronies become excessive, e.g. due to conflicting timing goals of partners, single dyad-members have been found to sacrifice their individual timing and to adapt to their partner’s timing, indicating a shift towards self-other integration for the sake of the joint action goal (MacRitchie et al. 2018). The current study focuses on subtle asynchronies that may favor self-other segregation. At the neural level, cortico-cerebellar and striato–thalamo–cortical loops are known to be involved in action timing and time perception, as well as in processes of sensorimotor error-correction in single performers (Brown et al. 2006; Molinari et al. 2007; Chen et al. 2009; Kornysheva and Schubotz 2011; Teki et al. 2011b; Rajendran et al. 2018; Cannon and Patel 2021). However, whether and how these regions contribute to the processing of temporal asynchronies during joint music performance and the balancing of self-other integration and segregation remains to date unclear.

Another factor that has been found to modulate the balance of self-other integration and segregation is the familiarity with the co-actor’s action or action style (Keller et al. 2014; Novembre and Keller 2014; Novembre et al. 2016; MacRitchie et al. 2018; Bolt and Loehr 2021; Liebermann-Jordanidis et al. 2021). For example, familiarity with a co-actor’s action on a supra-second level was found to facilitate a more precise prediction of their action goals during joint action (Sebanz and Frith 2004; Sebanz and Knoblich 2009; Keller et al. 2016). Contrarily, on a sub-second level, prior motor experience with an action performed by a co-actor was found to decrease the stability of interpersonal synchronization (Ragert et al. 2013) and to reduce mutual adaptation (Gugnowska et al. 2022), suggesting a favoring of stronger self-other segregation. The present study focuses on the effects of motor familiarity on the sub-second level, i.e. own motor knowledge of how to perform the partner’s part. It has been proposed that in this case, the micro-timing of the partner’s action is estimated based on an internal model of this action during joint performance (Wolpert et al. 2003; Lee and Noppeney 2011; Keller et al. 2014, 2016; Hadley and Pickering 2020). That is, the partner’s timing is internally anticipated by virtue of a motor simulation and auditory imagery based on one’s own motor repertoire and playing style, rather than by relying only on the actual partner-produced sensory feedback (Rizzolatti 2005; Wilson and Knoblich 2005; Keller et al. 2007; Knoblich and Sebanz 2008; Sebanz and Knoblich 2009; Pesquita et al. 2018). Note that timing idiosyncrasies in one’s own motor repertoire do not exactly match those of the partner, which can account for the shift towards stronger self-other segregation (Novembre et al. 2016) reflected in lower behavioral coupling (Ragert et al. 2013; Gugnowska et al. 2022).

Initial evidence for motor simulation of familiar actions (i.e. of actions oneself has practiced before) comes from studies showing robust responses in fronto-parietal motor regions during passive visual or auditory perception of motorically familiar compared to unfamiliar actions, as if observers were performing those actions themselves (Haueisen and Knösche 2001; Calvo-Merino et al. 2005, 2006; Bangert et al. 2006; D’Ausilio et al. 2006; Lahav et al. 2007; Hilt et al. 2020). More specific evidence for motor simulation during joint action comes from a series of transcranial magnetic stimulation (TMS) studies with pianists playing right-hand parts of piano duets. These pianists showed stronger behavioral interference in their right-handed performance when inhibitory TMS was applied to the primary motor cortex (M1) controlling their left hand, i.e. the hand used by their partner (Novembre et al. 2014; see also Hadley et al. 2015). This was taken to indicate that pianists engaged in motor simulation of their partner’s left-hand part when they had practiced it before. Moreover, motor evoked potentials (MEPs) in resting left-hand muscles were stronger when pianists performed the right-hand part together with a partner than alone (Novembre et al. 2012). Together, these studies strongly suggest that motor simulation processes mediate interpersonal coordination (for reviews, see Hadley and Pickering 2020; Bolt and Loehr 2021). Yet, the neural networks underlying these processes during real-time joint performance, and their contributions to the balancing of self-other integration and segregation remain to be shown.

A recent electroencephalography (EEG) study with piano duos investigated the interplay between motor familiarity and temporal compatibility in the balance of self-other integration and segregation (Novembre et al. 2016). It was found that right posterior EEG alpha power changed as a function of temporal compatibility: Highly synchronous joint performance was associated with alpha suppression, whereas slightly asynchronous interactions induced alpha enhancement. Notably, these alpha power modulations were only observed when pianists were familiar with their partner’s part. This result was taken to suggest that subtle auditory discrepancies due to slight asynchronies in performance might be processed differently by the brain depending on whether or not the actions of a partner belong to a performer’s motor repertoire. It was proposed that prior motor experience led to the development of an internal model of the other’s part that weighted self-other integration and segregation depending on interpersonal timing compatibility. Yet, this EEG-study did not address the neural networks underlying this interplay between internal models and interpersonal asynchrony, which was the goal of the current fMRI study.

We adapted the paradigm of Novembre et al. (2016) to be performed within an MRI setting. During the experiment, 1 pianist played the melody (right hand) of short duets on an MR-compatible piano in the MR-scanner, whereas the accompanying pianist played the bassline (left hand) on a MIDI-piano outside the scanner room. Pianists heard each other via headphones. The 2 × 2 experimental design contained 2 factors: The first factor—TEMPO—involved manipulating the temporal compatibility of pianists’ action feedback, i.e. the degree of synchrony between co-performers’ actions, in the range of milliseconds (reflected in higher mean absolute keystroke asynchronies between performers, see section “Materials and methods” for details; Keller et al. 2007; Van Der Steen et al. 2013; Keller 2014). On the behavioral level, we expected pianists to adapt less to their partners during subtly asynchronous compared to synchronous performance, i.e. to shift balance towards self-other segregation, reflected in reduced cross-correlations of partners’ inter-keystroke intervals (IKIs) at lag +1 and at lag −1 (see section “Materials and methods” for details; Fairhurst et al. 2013; Novembre et al. 2016; Koban et al. 2019). On the neural level, the cerebellum and the basal ganglia (BG) were hypothesized as plausible candidates for the detection of and adaptation to these subtle temporal asynchronies, due to the involvement of these subcortical structures in temporal and rhythm processing (Ivry et al. 2002; Grahn and Brett 2007; Chen et al. 2008a; Teki et al. 2011b; Rajendran et al. 2018; Cannon and Patel 2021), and audio-motor coordination (Zatorre et al. 2007; Chen et al. 2008b; Teki et al. 2011a).

The second factor—FAMILIARITY—involved manipulating whether pianists had or had not practiced each other’s part prior to participating in the experiment session (Novembre et al. 2012, 2014; Ragert et al. 2013; Hadley et al. 2015). Familiarity was expected to trigger motor simulation in right premotor cortex (PMC) and inferior parietal areas (Calvo-Merino et al. 2005, 2006; Casile and Giese 2006; Cross et al. 2006) associated with the familiar, albeit not performed, left-hand part (Novembre et al. 2012, 2014; Hadley et al. 2015). As these regions are structurally and functionally connected with auditory regions (Engel et al. 2014; Segado et al. 2018; Wollman et al. 2018), we additionally conducted functional connectivity analyses and expected to observe stronger audio-motor coupling during familiar, as opposed to unfamiliar, conditions. On the behavioral level, we expected lower synchronization stability (measured as the standard deviation of signed keystroke asynchronies between partners; Ragert et al. 2013) and weaker interpersonal coupling (measured as cross-correlations between partners’ inter-keystroke-intervals at lag 0; Konvalinka et al. 2010; Novembre et al. 2016) in familiar compared to unfamiliar conditions, indicating a shift towards stronger self-other segregation.

Finally, we examined the interaction between our 2 factors—TEMPO and FAMILIARITY—which is assumed to capture the neural balancing of self-other integration and segregation based on links between external sensory feedback and internal models (Novembre et al. 2016). Previous literature suggests several candidate structures possibly involved in this balancing act: The cerebellum has been discussed as a relevant structure for time and rhythm processing (as mentioned above) as well as for internal models of actions (Kawato and Gomi 1992; Wolpert et al. 1998; Ito 2005; Bastian 2006; Ishikawa et al. 2016; Johnson et al. 2019; Popa and Ebner 2019; Tanaka et al. 2020; Van Overwalle et al. 2020), making it a plausible candidate for the interplay of both factors. At the cortical level, the temporo–parietal junction (TPJ) and the precuneus (PCun) have been related to functions of self-other integration and segregation (Fairhurst et al. 2014; Heggli et al. 2021), compatible with the centro-parietal distribution of the EEG alpha power modulations in the study of Novembre et al. (2016).

## Materials and methods

### Participants

We tested 40 expert pianists (age range: 18–39 years, *M* = 25.25 years, *SD* = 5.30, 20 females, 4 left-handed) who were randomly allocated into 20 pairs (4 only-female, 4 only-male, 12 mixed-gender pairs, mean age difference between partners: 5.30 years, *SD* = 4.43). All pianists had played the piano for an average of 17.18 years (*SD* = 5.86, range: 8–32 years), had started playing at a mean age of 7.70 years (*SD* = 3.07, range: 4–16 years), and were musically active at the time of testing (weekly practice: *M* = 8.73 h *SD* = 9.69, range: 2–50 h). Handedness was assessed with the Edinburgh Handedness Inventory (Oldfield 1971). One participant who was not able to perform the musical pieces in the pre-experimental test session (see below) was excluded from further participation and replaced by another pianist. All pianists had normal or corrected-to-normal vision, reported normal hearing, no neurological or psychiatric history, and no contraindication for MRI. The pianists were naïve to the purpose of the study and received monetary compensation for their participation. The study was approved by the ethics committee of the University of Leipzig (016-15-26012015) and was conducted following the guidelines of the Declaration of Helsinki. All pianists provided written informed consent.

### Materials and pre-experimental training at home

**Fig. 1:**
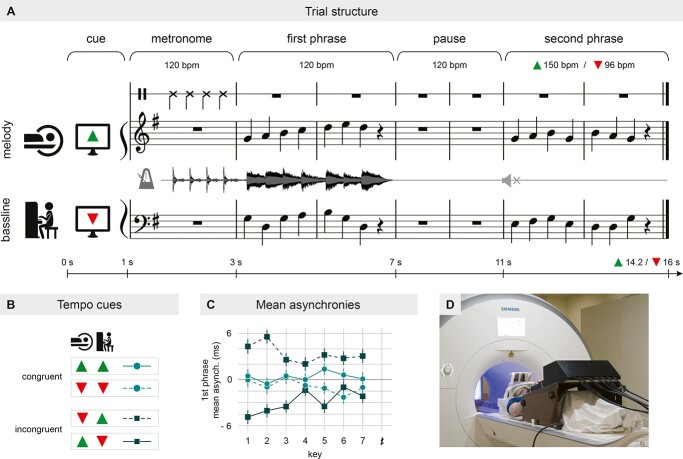
Experimental design. A) Experimental setup and trial structure with 1 of the 8 pieces as an example; B) the 4 combinations of tempo instructions for the second phrase tempo, which result in 2 TEMPO conditions: Congruent (up-up, down-down) and incongruent (up-down, down-up); C) signed interpersonal keystroke asynchronies in the first phrase, averaged for each type of tempo instruction. The higher asynchronies in incongruent (compared to congruent) trials indicate that pianists already anticipated the tempo change at the beginning of the first phrase; and D) pianist with MR-piano, about to enter the MR-scanner.

Eight modified 6-bar-excerpts of 2-voiced chorales by J.S. Bach served as musical material in the present experiment (see Fig. 1). Each chorale was composed of 2 musical phrases of 2 bars each, separated by a pause of 2 bars. Both phrases consisted of 7 crotchets (quarter notes) and a crotchet pause. All pieces contained a melody for the right hand and a bassline for the left hand, which were matched in difficulty both across hands and conditions by controlling the interval sizes in both voices. Pianists received the scores of these pieces and 2 metronome-files (see below) for rehearsal at home, ~2 weeks prior to the experiment.

In order to manipulate pianists’ motor FAMILIARITY with their partner’s part, we varied which scores pianists received for practice. For 4 pieces, they received the full scores and practiced both their own and their partner’s part (melody and bassline), so that both parts were familiar (condition abbreviated as F). For the other 4 pieces, they received partial scores to practice only the melody (two pieces) or only the bassline (two pieces), complementarily within a pair, so that their partner’s respective part was unfamiliar (U). The pieces for which both parts were practiced were counterbalanced across the group. Later, during the experiment, one pianist played the practiced melodies, the other the practiced basslines of the pieces, while being familiar or unfamiliar with the respective other part.

To manipulate the TEMPO compatibility of pianists’ actions, they were asked to practice all pieces with a predefined tempo change in the second phrase. As shown in Fig. 1A, the first phrase (bars 1–2) and the pause (bars 3–4) had to be performed at a tempo of 120 beats per minute (bpm), the second phrase (bars 5–6) had to be played either at a faster (150 bpm) or a slower tempo (96 bpm) as cued at trial onset. Pianists practiced these tempo changes with the help of 2 audio-files with metronome-clicks. Each metronome-file consisted of 7 bars with 4 clicks each (including 1 preparatory bar), at the required tempo. Pianists learned all pieces by heart in both tempo variations. Later, during the experiment and unbeknownst to the pianists, they were cued to perform either congruent or incongruent tempo changes (Fig. 1B). That is, both pianists speeded up or slowed down in the second phrase (congruent; C), or one pianist speeded up and the other slowed down, and vice versa (incongruent; I). As demonstrated by Novembre et al. (2016), incongruent tempo instructions induce subtle interpersonal keystroke asynchronies already during the first phrase (see Fig. 1C), i.e. naturally modulate the temporal compatibility of the partners’ actions.

### Pre-experimental test session

Approximately 1 week prior to the experiment, each pianist was invited for a solo session to familiarize themselves with playing the piano in supine position and to test their ability to perform the pieces as well as the tempo changes. Therefore, pianists were first asked to perform all pieces as rehearsed, from memory sitting on a MIDI keyboard (M-Audio Keystation 49e, inMusic GmbH, Ratingen, Germany), without seeing the scores and without a metronome guiding the tempo change. Half of the pieces had to be played with changes to the fast tempo, and the other half with changes to the slow tempo in the second phrase. Only pianists who were able to perform all 8 pieces and both tempo changes accurately by heart took part in the main experiment (see section “Participants”). To practice playing the piano in supine position, these pianists were then positioned in a mock MR-scanner with the MIDI-piano on their lap, where they performed the rehearsed melodies of 4 pieces with their right hand. In 2 of these pieces, they were familiar with the basslines and in the other 2, they were unfamiliar with the basslines. Subsequently, they again sat down in front of the MIDI keyboard and played the rehearsed basslines of the other 4 pieces with their left hand. In 2 of these pieces, they were familiar with the melodies and in the other 2, they were unfamiliar with the melodies. The scores of the to-be-played part (melody or bassline) were displayed on a computer monitor (visible via a mirror system in the mock scanner). Each melody and bassline was performed twice, once with each type of tempo change, resulting in a total of 16 trials during the test session. This solo session took ~2 h per pianist.

### Experimental procedure

The fMRI experiment consisted of 2 consecutive scanning sessions separated by a 30-min break. In the first session, pianist A played the piano in the MR-scanner in duet with accompanying pianist B who played outside the scanner room. They swapped places in the second session.

Upon their arrival, the 2 pianists were introduced to each other and were left alone for ~10 min to ensure that pianists knew with whom they were interacting during performance. Afterwards, they were positioned in the MR-scanner and the adjacent room respectively. First, they had the opportunity to familiarize themselves with the setup and with each other’s playing skills and style by playing freely together whatever came to their minds (except for the experimental pieces). This served to relax the MR-pianist in the uncommon position and took about 5 min. Thereafter, 16 practice trials with the experimental pieces ensured that the pianists were able to play the rehearsed pieces together, that they had understood the instructions and heard each other’s performances well. Switching hands between trials was expected to induce head movements, therefore the pianist inside the MR-scanner always used the right hand (playing the melody), whereas the other pianist played with the left hand (the accompanying bassline).

During each session, the pianists played 4 of the 8 practiced pieces in the same position (supine or sitting upright). The pieces per session were counterbalanced across pairs. Pianists completed 128 trials, 32 in each of the 4 conditions of the 2 × 2 factorial design. Each trial started with a visual cue (1,000 ms) that indicated whether to play the fast (green triangle pointing upward) or the slow tempo in the second phrase of the piece (red triangle pointing downwards; see Fig. 1A). In half of the trials, pianists received the same cue (both green or both red; congruent tempo), in the other half they saw opposite cues (one green, the other red; incongruent tempo; Fig. 1B). Each pianist saw only 1 cue and was not aware of the cue presented to the other pianist. After the cue, the musical scores of the pianist’s respective part (but not the partner’s part) appeared on screen and 4 metronome beats were presented at a tempo of 120 bpm (lasting 2,000 ms in total) after which pianists were supposed to start playing. Pianists were required to play the first 2 bars at 120 bpm, to pause during bars 3 and 4 (also at 120 bpm), and then to complete the last 2 bars at the tempo indicated by the visual cue at trial beginning, leading to congruent or incongruent new tempi between co-performers. To leave pianists naïve to this manipulation, both pianos were muted (automatically by the experimental script) during the second phrase. Importantly, the anticipated tempo change in the second phrase induces slight deviations in pianists’ timing already in the first phrase, despite the joint tempo of 120 bpm, leading to lower behavioral synchrony in incongruent (compared to congruent) trials (see Fig. 1C; Novembre et al. 2016). To (mis)lead pianists into believing that they always received the same tempo instructions, the first 8 practice trials before the experiment were all congruent and not muted during the second phrase. Trials lasted between 14.2 s (fast tempo) and 16 s (slow tempo) and were separated by a jittered inter-trial-interval of 3–9 s during which a fixation cross was shown. Scanning duration of 1 session was ~45 min.

After completion of the first session, pianists took a 30-min break before the experiment was repeated with the remaining 4 pieces, with pianist B in the scanner and pianist A outside. The whole experimental procedure, including preparation time, 2 sessions and breaks, took ~5 h per pair.

### Experimental setup and data acquisition

Behavioral data were acquired via a custom-made 27-key MR-compatible MIDI-piano (Julius Blüthner Pianofortefabrik GmbH, Leipzig, Germany; see Fig. 1D), with auditory feedback received via MR-compatible in-ear headphones (Sensimetrics, MR confon GmbH, Magdeburg, Germany). The piano was placed on a slightly tilted wooden stand clipped into the scanner bed over the pianist’s lap. An MR-compatible camera (12M camera, MRC Systems, Heidelberg, Germany) was placed on top of the piano to record the pianist’s finger movements. A double mirror system mounted on the head coil allowed the pianist to see both the piano and the visual stimuli projected onto a screen at the head-end of the MR-scanner. Pianist B was seated in a separate room at a Yamaha Clavinova CLP150 on top of which a 16″ Sony Trinitron Multiscan E220 monitor (100-Hz refresh rate) was placed for presentation of visual stimuli. Sound was delivered via DT 770 PRO, 250 Ohms headphones (beyerdynamic, Heilbronn, Germany). The audio-output of both pianos was fed into and mixed through an McCrypt SA-101U USB DJ-mixer (Renkforce, Conrad Electronic SE, Hirschau, Germany) that was located in the control room where the experimenters were seated. The experiment was controlled with Presentation software (Version 16.5, Neurobehavioral Systems, Inc., Berkeley, CA, United States) and custom Python programs to record the MIDI output of the pianos.

MR-data were collected at the Max Planck Institute for Human Cognitive and Brain Sciences, Leipzig, in a 3-Tesla Siemens Skyra magnetic resonance scanner (Siemens AG, Erlangen, Germany) using a 32-channel head coil. Functional images were acquired with a whole-brain multi-band echo-planar imaging sequence (EPI; *TR* = 2,000 ms, *TE* = 22 ms, multi-band acceleration factor = 3, 60 axial slices in interleaved order, voxel size = 2.5 mm^3^, 10% inter-slice gap, flip angle = 80°, field of view = 204 mm; Feinberg et al. 2010; Moeller et al. 2010). Anatomical T1-weighted images were acquired with a whole-brain magnetization-prepared rapid acquisition gradient echo sequence (MPRAGE; *TR* = 2,300 ms, *TE* = 5.52 ms, 176 sagittal slices, voxel size = 1 mm^3^, flip angle = 9°, field of view = 256 mm; Mugler and Brookeman 1991).

### Behavioral data analysis

In a first step, all trials in which at least 1 of the pianists played a wrong key were excluded. Trials in which pianists did not change the tempo correctly were also discarded, i.e. trials in which the 6 IKIs of a pianist in the second phrase showed a better fit with IKIs expected for a tempo other than instructed. The goodness of fit was assessed by comparing the mean squared differences between the played IKIs and the IKIs expected for 150 bpm (fast; 6 times 400 ms), 96 bpm (slow; 6 times 625 ms), or 120 bpm (no tempo change; 6 times 500 ms). Furthermore, trials with technical errors were eliminated. Data of 2 pianists (one pair) had to be discarded because of a technical failure (fMRI data were analyzed), leading to a behavioral sample size of *N* = 38. Visual inspection of the videos of this pair showed that they made only very few errors in total (18 out of 256 trials), which is why their neural data were retained in the fMRI analysis. As a last step, trials with the 1% longest and 1% shortest IKIs in the first phrase (i.e. outlier IKIs across pianists and conditions) were eliminated to account for rare rhythmic deviations. Final mean trial numbers per pair per condition were as follows: familiar–congruent (FC): 19.26; familiar–incongruent (FI): 18.08; unfamiliar–congruent (UC): 20.00; and unfamiliar–incongruent (UI): 18.95. All behavioral analyses were conducted in R version 3.5.1 (R Core Team 2017), including the package “ez” (Lawrence 2016) for analyses of variance (ANOVAs).

Analyses focused on the first phrase of the pieces during which pianists heard each other. In order to assess the accuracy and stability of pianists’ behavioral synchronization during performance, keystroke asynchronies between players were calculated by subtracting the time of each keystroke of the accompanying pianist from the time of the respective keystroke of the pianist playing the melody inside the MR-scanner (Keller et al. 2007; Ragert et al. 2013). To account for possible differences in difficulty between pieces and keystroke positions (i.e. notes 1–7 in a piece), asynchronies were mean-centered by subtracting the mean keystroke asynchrony separately for each keystroke position and piece (Wing et al. 2014). Mean keystroke asynchronies after mean-centering are visualized in Fig. 1C, averaged over each of the 4 tempo instructions (i.e. both pianists speeded up, both slowed down, the MR-pianist speeded up and accompanying pianist slowed down, and vice versa). In order to evaluate the accuracy of interpersonal keystroke timing, asynchronies were averaged across keystrokes 1–7, separately for each of the 4 tempo instructions. Afterwards, absolute values of these mean asynchronies were averaged for each of the 4 experimental conditions (FC, FI, UC, and UI). To estimate the stability of interpersonal keystroke timing, standard deviations (*SD*) of signed asynchronies were computed across keystrokes 1–7 and then averaged across trials per condition. Mean absolute asynchronies and mean *SD*s of the asynchronies were statistically compared in separate 2 × 2 repeated-measures (rm) ANOVAs with the factors FAMILIARITY (familiar/unfamiliar with the partner’s part) and TEMPO (congruent/incongruent tempo instructions).

In order to evaluate to what extent pianists adapted the timing of their performance to that of their partners’ timing, IKIs of both pianists were cross-correlated at lags 0, +1, and −1 (Goebl and Palmer 2009; Konvalinka et al. 2010, 2014). Coefficients at lag 0 were taken as index for interpersonal coupling in real-time. Lagged cross-correlations were calculated taking the IKIs of the MR-pianist as reference relative to which the IKIs of the partner were shifted. This and the fact that both pianists had constant roles throughout the experimental session (i.e. always played the melody or the bassline) allowed us to evaluate how strongly the MR-pianist adapted to the preceding IKIs of the partner (lag −1), as well as how strongly the partner adapted to the MR-pianist in the following IKIs (lag +1). Coefficients (Fisher *z*-transformed) were averaged across trials, separately for each of the 4 conditions, and statistically evaluated. Coefficients at lag 0 were compared in a 2 × 2 rmANOVA with factors FAMILIARITY and TEMPO, and coefficients at lag +1 and −1 were evaluated in a 2 × 2 × 2 rmANOVA with factors FAMILIARITY, TEMPO, and LAG (lag +1/lag −1).

### FMRI data analysis

FMRI data were analyzed using SPM12 (Wellcome Trust Centre for Neuroimaging, London, United Kingdom) in Matlab version 9.3 (R2017b). Data preprocessing followed the standard pipeline in SPM12 and included slice-time correction, realignment and unwarping, segmentation, co-registration of the functional, and anatomical images, normalization into the Montreal Neurological Institute (MNI) stereotactic space with resampling to 2-mm^3^ voxel size, and smoothing using a Gaussian kernel of 8-mm full width at half maximum (FWHM). One pianist was excluded from further analyses due to perturbed structural image preprocessing (the behavioral data were retained), which resulted in 39 pianists for the fMRI analysis.

On the first level, preprocessed data of each pianist were analyzed with a General Linear Model comprising 4 predictors modeled using a finite impulse response function with a length of 9 volumes covering 18 s, i.e. the maximal trial length of 16 s plus one volume to account for trials in which pianists played more slowly than expected (see Fig. 1). Onsets were modeled relative to the onset of the metronome, with a lag of 4 s to account for the lag of the hemodynamic response. It was assumed that the first volume mainly reflected activity associated with hearing the metronome, whereas volumes 2 and 3 should reflect activity evoked by the joint performance during the first phrase, relevant for the present analysis. Trials were assigned to the 4 predictors depending on whether or not pianists were familiar with their partner’s part and whether they received congruent or incongruent tempo instructions. Resulting predictors were labeled familiar–congruent (FC), familiar–incongruent (FI), unfamiliar–congruent (UC), and unfamiliar–incongruent (UI). In addition, 6 motion parameters were entered as covariates of no interest to control for subtle head movements. Baseline contrasts of volumes 2 and 3 were calculated, separately for each of the 4 conditions, for use in the second level group analysis.

On the group level, the 4 conditions were compared in a 2 × 2 flexible factorial design with the factors FAMILIARITY and TEMPO. We calculated the main effect of FAMILIARITY (familiar > unfamiliar *T*-contrast) to identify brain regions involved in the motor simulation of the partner’s part. The main effect of TEMPO (incongruent > congruent *T*-contrast) was computed to identify brain areas involved in compensating for asynchronies in joint performance. Finally, an interaction between FAMILIARITY and TEMPO was calculated [(FI > FC) > (UI > UC)] to elucidate which regions are particularly sensitive to tempo discrepancies when pianists were familiar with the partner’s part.

In a follow-up analysis, we also explored the main effect of FAMILIARITY (F > U) in the second phrase of the trials. This was meant to elucidate whether effects from the first phrase would replicate without sound, i.e. without tangible social contact between partners. We note, however, that these results should be interpreted cautiously, because the second phrase is not fully independent from the first phrase due to the sluggish BOLD response, and because the 2 phrases differ in performance tempo (96 or 150 bpm vs. 120 bpm) and duration (5 s or 3.2 s vs. 4 s).

The statistical threshold was estimated based on a nonparametric Monte Carlo simulation (Slotnick et al. 2003; code available at https://drive.google.com/file/d/16HVUD-PZaEpwHoZE99YXDxhcuLawjW7O/view?usp=sharing), addressing emerging concerns of balancing whole-volume type I and type II errors (Slotnick 2017; Noble et al. 2020). This simulation implemented in Matlab (1,000 iterations, no volume mask) suggested a cluster extent threshold of at least 33 resampled voxels and a voxel-level uncorrected *P*-value of 0.001 to ensure a whole-volume type I error probability smaller than 0.05. Anatomical labels were identified using the Harvard-Oxford cortical and subcortical structural atlases in FSL version 5.0.9 (Analysis Group, FMRIB, Oxford, United Kingdom).

### Psychophysiological interaction analysis

To identify task-dependent changes in connectivity related to the pianist’s motor simulation of their partner’s action, we performed a psychophysiological interaction (PPI) analysis. In order to define volumes of interest (VOIs), a sphere with 15-mm radius was centered on the group-level activation peaks from the factorial analysis, in right pre- and postcentral gyri [PrCG: 26, −12, 60], [PoCG: 44, −30, 46], and left PoCG [−44, −22, −62] (while left precentral gyrus did not yield enough data points for a PPI analysis). Within these spheres, each pianist’s individual local activation peak was identified, and voxels within a radius of 6 mm around that peak that were active at *P* < 0.01 (uncorrected) were defined as VOIs. Pianists without local peak in the 15-mm sphere were excluded from further analyses, resulting in *N* = 27 pianists in the PPI of right PrCG, *N* = 28 for right PoCG, and *N* = 31 for left PoCG. The mean time course of the fMRI signal changes in each VOI was then extracted and multiplied by a regressor representing the experimental conditions (familiar/unfamiliar). This interaction term of source signal and experimental treatment was the regressor of interest in the PPI model. In addition, the mean deconvolved source signal of the VOI and a FAMILIARITY regressor were included as covariates of no interest. Group-level significance was assessed by means of 1-sample *t*-tests against zero. The statistical threshold was *P* < 0.001 at voxel-level followed by cluster-level FWE correction at *P* < 0.05.

## Results

### Behavioral data

#### Synchronization accuracy: absolute asynchronies

**Fig. 2:**
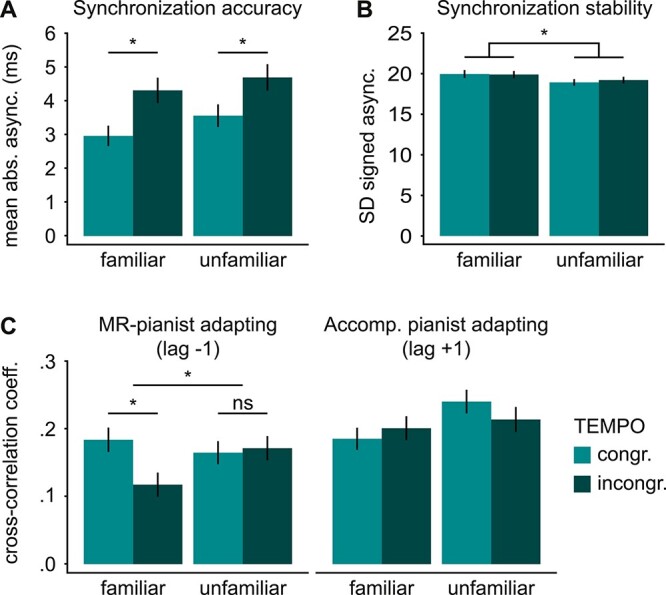
Behavioral results (first phrase). A) Mean absolute asynchronies between pianists showed a main effect of TEMPO. Lower values in the congruent TEMPO condition indicate higher synchronization accuracy; B) the SD of signed asynchronies showed a main effect of FAMILIARITY. Lower values in the unfamiliar condition indicate higher synchronization stability; and C) cross-correlation coefficients at lag −1 and lag +1. Coefficients at lag −1 indicate how strongly the MR-pianists adapted to their partner. They show a FAMILIARITY × TEMPO interaction reflecting a TEMPO effect in familiar (but not unfamiliar) conditions. Coefficients at lag +1 indicate how strongly the accompanying pianist adapted to the MR-pianist. Higher coefficients indicate stronger adaptation. ^*^  *P* < 0.05.

Synchronization accuracy in the first phrase was analyzed by entering mean absolute asynchronies into a 2 × 2 ANOVA with the repeated measures factors FAMILIARITY (familiar/unfamiliar) and TEMPO (congruent/incongruent). The analysis revealed a main effect of TEMPO, showing that asynchronies were higher in trials with incongruent (mean ± 1 standard error of the mean, *SEM*: 4.51 ± 0.271 ms), compared to congruent (3.27 ± 0.226 ms) tempo instructions for the second phrase (see Fig. 2A). This effect provides evidence for the effectiveness of the paradigm, as the purpose of the TEMPO manipulation was to influence the degree of interpersonal synchrony, i.e. temporal compatibility of action feedback during the first phrase. No other significant effects were found (for statistical details, see Table 1).

**Table 1:** ANOVA results for behavioral synchronization during the first phrase.

	Mean absolute asynchronies			*SD* of signed asynchronies		
	*F*(1,37)	*P*	*n_p_^2^*	*F*(1,37)	*P*	*n_p_^2^*
Familiarity	1.82	0.185	0.05	8.90	**0.005**	0.19
Tempo	9.19	**0.004**	0.20	0.20	0.654	0.01
Familiarity × Tempo	0.12	0.728	< 0.01	0.46	0.502	0.01

*Note:* Significant *P*-values are shown in bold.

#### Synchronization stability: standard deviation of asynchronies

Synchronization stability was analyzed in a 2 × 2 ANOVA on the *SD* of asynchronies, revealing a main effect of FAMILIARITY. The *SD* of asynchronies was higher in familiar (19.93 ± 0.325 ms), compared to unfamiliar conditions (19.07 ± 0.288 ms; see Fig. 2B). This shows that pianists’ interpersonal keystroke asynchronies were less variable, i.e. their synchronization stability was higher, in unfamiliar compared to familiar conditions. No other significant effects were found (for statistical details, see Table 1).

#### Interpersonal adaptation: cross-correlation coefficients

To analyze the adaptation of both pianists to each other’s performance timing, a 3-way ANOVA with the factors FAMILIARITY, TEMPO, and LAG (lag +1/lag −1) was calculated on cross-correlation coefficients between pianists’ IKIs (see Fig. 2C and Table 2). The analysis revealed a main effect of LAG, with higher cross-correlation coefficients at lag +1 (0.211 ± 0.009) than lag −1 (0.160 ± 0.009). This indicates that the accompanying pianist adapted more strongly to the MR-pianist than vice versa. The analysis also returned a significant 3-way interaction. No other significant effects were found (for statistical details, see Table 2).

**Table 2:** Three-way ANOVA results for behavioral adaptation during the first phrase.

Cross-correlations	Lag −1 and Lag +1		
	*F*(1,37)	*P*	*n_p_^2^*
Familiarity	2.07	0.158	0.05
Tempo	2.99	0.092	0.07
Lag	4.12	**0.050**	0.10
Familiarity × Tempo	1.12	0.298	0.03
Tempo × Lag	1.51	0.228	0.04
Familiarity × Lag	0.22	0.638	0.01
Familiarity × Tempo × Lag	5.51	**0.024**	0.13

*Note:* Lag − 1 indicates adaptation of the MR-pianist to their partner. Lag + 1 indicates adaptation of the accompanying pianist to the MR-pianist. Significant *P*-values are shown in bold.

The 3-way interaction was then split by the factor LAG, resolving into separate FAMILIARITY × TEMPO ANOVAs for lag −1 and lag +1 (see Table 3). This separation was motivated by the assumption that cross-correlations at lag −1 reflect the adaptation of the MR-pianists to their partner, whereas cross-correlations at lag +1 reflect the adaptation of the accompanying pianist (see section “Materials and methods”). The ANOVA on cross-correlations at lag −1 yielded a significant main effect of TEMPO and a FAMILIARITY × TEMPO interaction (no main effect of FAMILIARITY). Subsequent *t*-tests showed a significant difference between congruent and incongruent trials in familiar conditions (FC vs. FI: *t*(37) = 2.99, *P* = 0.005, *d* = 0.49), whereas no such effect was found in unfamiliar conditions (UC vs. UI: *t*(37) = −0.25, *P* = 0.804, *d* = 0.04). More precisely, cross-correlation coefficients at lag −1 were significantly lower in familiar-incongruent (FI: 0.118 ± 0.018), compared with familiar-congruent trials (FC: 0.184 ± 0.018). This indicates that the MR-pianists’ adaptation to their partner was influenced by the TEMPO manipulation in familiar, but not unfamiliar conditions. The ANOVA on cross-correlations at lag +1 (reflecting the accompanying pianist’s adaptation to the MR-pianist) showed no significant effects (see Table 3 for statistical details).

**Table 3:** Two-way ANOVA results for behavioral adaptation during the first phrase.

Cross-correlations	Lag −1			Lag 0			Lag +1		
	*F*(1,37)	*P*	*n_p_^2^*	*F*(1,37)	*P*	*n_p_^2^*	*F*(1,37)	*P*	*n_p_^2^*
Familiarity	0.54	0.468	0.01	0.00	0.970	< 0.01	1.75	0.194	0.05
Tempo	4.60	**0.039**	0.11	2.00	0.165	0.05	0.07	0.799	< 0.01
Familiarity × Tempo	4.35	**0.044**	0.11	0.36	0.553	0.01	0.92	0.345	0.02

*Note:* Lag − 1 indicates adaptation of the MR-pianist to their partner. Lag 0 indicates mutual adaptation. Lag + 1 indicates adaptation of the accompanying pianist to the MR-pianist. Significant *P*-values are shown in bold.

Finally, to analyze the degree of mutual adaptation, a 2 × 2 ANOVA with the factors FAMILIARITY and TEMPO was calculated on the cross-correlations of pianists’ IKIs at lag 0. No significant differences were found between the experimental conditions (see Table 3 for statistical details).

### fMRI data

**Fig. 3:**
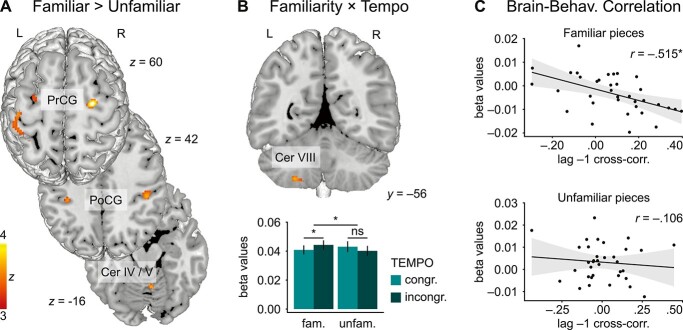
Neural results (first phrase) and correlations with behavior. A) Comparison of brain activation for familiar > unfamiliar conditions; B) brain area showing a FAMILIARITY × TEMPO interaction [(FI > UC) > (FC > UI)], including beta values at cluster peak (extracted with rfxplot); and C) brain-behavior correlation between cerebellar (Cer VIII) activation changes (incongruent minus congruent) and corresponding changes in lag −1 cross-correlation coefficients (behavioral adaptation of the MR-pianist to their partner), separately for familiar and unfamiliar conditions. Threshold: *P*-voxel < 0.001; cluster extent ≥33 re-sampled voxels corresponding to *P*-cluster < 0.05 according to Slotnick et al. (2003).

Figure 3A presents the results of the flexible factorial analysis of the first phrase. It revealed a main effect of FAMILIARITY in sensorimotor areas showing stronger activity when pianists played pieces in which they were motorically familiar (compared to unfamiliar) with their partner’s part. These areas comprised bilateral premotor and somatosensory regions in precentral and postcentral gyri (PrCG and PoCG), as well as left superior parietal lobule (SPL) and cerebellar sensorimotor regions IV and V (see also Table 4). No such effects were found in an analogous contrast in the (muted) second phrase, suggesting a dependency of these results on auditory information and social interaction between partners. No effect was found in the unfamiliar > familiar contrast.

**Table 4:** Results of the fMRI flexible factorial analysis.

Region	Hem.	BA	*k*	*z*-value	MNI coordinates		
					*x*	*y*	*z*
Main effect FAMILIARITY (Familiar > Unfamiliar)							
**Precentral gyrus (PrCG)**	**R**	**6**	**176**	**3.99**	**26**	**−12**	**60**
		6		3.68	30	−16	72
**Precentral gyrus (PrCG)**	**L**	**6**	**36**	**3.52**	**−32**	**−10**	**68**
		6		3.26	−28	−6	60
**Postcentral gyrus (PoCG)**	**R**	**2**	**147**	**3.80**	**44**	**−30**	**46**
		3		3.45	46	−24	38
**Postcentral gyrus (PoCG)**	**L**	**4**	**156**	**3.71**	**−44**	**−22**	**62**
		3		3.61	−44	−34	64
Superior parietal lobule (SPL)		7		3.40	−34	−48	66
**Postcentral gyrus (PoCG)**	**L**	**40**	**41**	**3.48**	**−34**	**−34**	**42**
**Cerebellum (V)**	**R/L**	**—**	**38**	**3.58**	**4**	**−56**	**−16**
Cerebellum (IV)		—		3.14	−2	−52	−6
**White matter**	**R**	**—**	**206**	**3.88**	**40**	**−46**	**2**
		—		3.51	28	−54	26
		—		3.43	32	−56	18
**White matter**	**L/R**	**—**	**52**	**3.36**	**−10**	**−34**	**12**
		—		3.27	2	−34	12
Interaction FAMILIARITY × TEMPO							
**Cerebellum (VIII)**	**L**	**—**	**36**	**3.60**	**−26**	**−56**	**−50**
Familiar–Incongruent > Familiar–Congruent (paired-samples *t*-test)							
**Cerebellum (VIII)**	**L**	**—**	**41**	**3.69**	**−20**	**−52**	**−52**

*Note:* Peak voxels in clusters are shown in bold. Threshold: *P*-voxel < 0.001; cluster extent ≥ 33 re-sampled voxels corresponding to *P*-cluster < 0.05 according to Slotnick et al. (2003). *Abbreviations:* BA, Brodmann area; *k*, cluster size (number of voxels); Hem., hemisphere; L, left hemisphere; R, right hemisphere.

No main effect of TEMPO was found. That is, no region showed significantly stronger activity in the incongruent > congruent or the congruent > incongruent contrast.

Figure 3B visualizes a significant FAMILIARITY × TEMPO interaction (see also Table 4) in left cerebellar motor region VIII. Parameter estimates show that only during familiar (not unfamiliar) trials, this region was significantly more active with incongruent compared to congruent tempo instructions. This effect was confirmed by whole-brain paired-samples *t*-tests in SPM comparing FI > FC, and UI > UC.

### Brain-behavior correlations

To estimate the relationship between the neural and behavioral results, we extracted parameter estimates from individual brain data at the group-peak of the FAMILIARITY × TEMPO interaction (left cerebellum [−26, −56, −50]), and at the 3 coordinates used for the PPI analyses based on the main effect of FAMILIARITY (right PrCG [26, −12, 60], right PoCG [44, −30, 46], and left PoCG [−44, −22, 62]). These values were correlated with the behavioral measures that showed a FAMILIARITY × TEMPO interaction or main effect of FAMILIARITY, respectively, i.e. cross-correlation coefficients at lag −1 (FAMILIARITY × TEMPO coordinate) and *SD* of asynchronies (FAMILIARITY coordinates). Following Bonferroni-correction for multiple comparisons, results were considered significant at *P* < 0.0125.

In order to estimate brain-behavior correlations for the main effect of FAMILIARITY, differences between familiar and unfamiliar trials were calculated for the parameter estimates at the 3 PrCG/PoCG coordinates and for the *SD* of asynchronies. None of the 3 Pearson correlations was significant (all *P*s > 0.1).

Brain-behavior correlations of the FAMILIARITY × TEMPO interaction were calculated separately for familiar and unfamiliar trials, and then compared. Therefore, cerebellar parameter estimates and lag −1 cross-correlation coefficients in congruent trials were subtracted from incongruent trials, separately for familiar (i.e. FI–FC) and unfamiliar conditions (i.e. UI–UC). Pearson correlations for familiar conditions showed a significant negative correlation (*r* = −0.515, *P* = 0.002, *R*^2^ = 0.265), which was not the case for unfamiliar conditions (*r* = −0.106, *P* = 0.543, *R*^2^ = 0.011). The 2 correlations differed significantly from each other, as indicated by a Fisher’s *Z*-test on Fisher’s *r*-to-*z* transformed data (*z* = −1.909, *P* = 0.028; https://www.psychometrica.de/correlation.html).

### Functional connectivity of brain regions related to familiarity

**Fig. 4:**
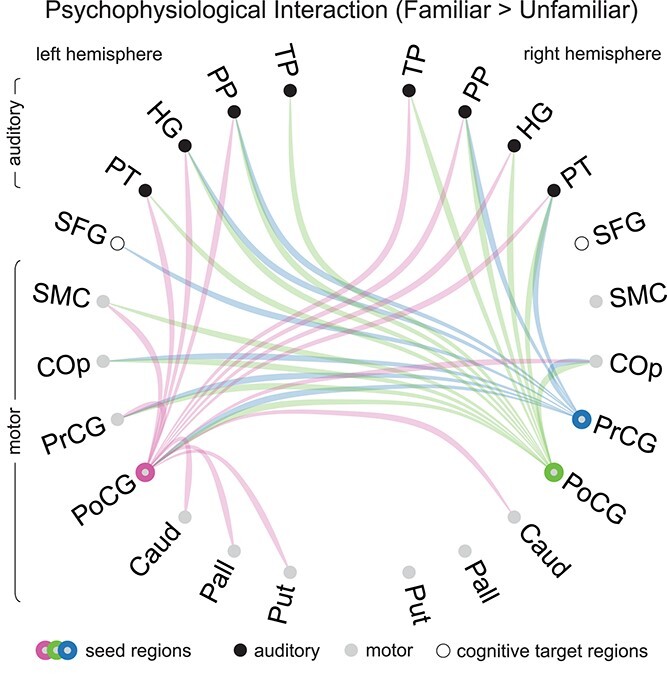
Functional connectivity. A psychophysiological interaction analysis with seeds in right precentral (PrCG) gyrus (blue) and bilateral postcentral gyri (PoCG; pink and green) revealed stronger audio-motor connectivity in familiar than unfamiliar conditions. Auditory areas (black dots): Planum temporale (PT); Heschl’s gyrus (HG); planum polare (PP); temporal pole (TP). Motor areas (gray dots): Central operculum (COp); supplementary motor cortex (SMC); caudate (Caud); pallidum (Pall); and putamen (Put). Cognitive areas (white dots): superior frontal gyrus (SFG).

Three regions that were more active in familiar compared to unfamiliar conditions (right PrCG and PoCG, left PoCG) served as seed regions in PPI analyses. All these regions showed increased connectivity to bilateral auditory regions when pianists were familiar with their partner’s part, including planum temporale (PT), planum polare (PP), Heschl’s gyrus (HG), and temporal pole (TP). In addition to that, right PrCG showed connectivity increases to left PrCG, PoCG, superior frontal gyrus (SFG), and bilateral central operculum (COp). Right PoCG showed connectivity increases to left PoCG, PrCG, supplementary motor cortex (SMC), and bilateral COp. Finally, left PoCG showed connectivity increases to left PrCG, SMC, pallidum and putamen, right COp, and bilateral caudate nucleus (see Fig. 4 and Table 5). Taken together, motor-familiarity was associated with enhanced audio-motor connectivity.

**Table 5:** Results of the functional connectivity analysis (PPI).

Region	Hem.	BA	*k*	*z*-Value	MNI coordinates		
					*x*	*y*	*z*
Seed in right precentral gyrus [26, −12, 60]							
**Precentral gyrus (PrCG)**	**L**	**6**	**1,290**	**3.77**	**−54**	**0**	**44**
		6		3.70	−44	−8	56
Postcentral gyrus (PoCG)		4		3.62	−32	−30	68
**Planum temporale (PT)**	**R**	**42**	**1,129**	**3.85**	**60**	**−28**	**16**
Central operculum (COp)		48		3.81	56	−4	8
Planum polare (PP)		22		3.69	44	−20	−4
**Planum polare (PP)**	**L**	**22**	**884**	**4.11**	**−48**	**0**	**−6**
Heschl’s gyrus (HG)		41/42		3.69	−38	−26	4
Central operculum (COp)		48		3.52	−54	−2	12
**Superior frontal gyrus (SFG)**	**L**	**6**	**711**	**4.04**	**−12**	**−4**	**74**
		6		3.74	−20	−4	70
		6		3.65	−12	−8	58
Seed in right postcentral gyrus [44, −30, 46]							
**Heschl’s gyrus (HG)**	**R**	**41/42**	**1,981**	**4.40**	**44**	**−16**	**10**
Central operculum (COp)		48		4.11	56	−6	8
Posterior superior temporal gyrus (pSTG)		22		4.07	66	−22	4
Planum temporale (PT)		42		3.79	60	−30	16
**Heschl’s gyrus (HG)**	**L**	**41/42**	**1,853**	**4.80**	**−44**	**−16**	**10**
Planum polare (PP)		22		4.39	−48	2	−4
Central operculum (COp)		48		4.25	−50	2	4
Temporal pole (TP)		38		3.82	−50	8	−16
Planum temporale (PT)		42		3.78	−54	−34	14
**Precentral gyrus (PrCG)**	**L**	**6**	**1,438**	**4.13**	**−52**	**0**	**46**
Precentral/postcentral gyrus (PrCG/PoCG)		6/4		4.07	−48	−14	44
Postcentral (PoCG)		4		3.78	−36	−30	66
**Supplementary motor cortex (SMC)**	**L**	**6**	**554**	**3.76**	**−8**	**2**	**74**
		6		3.74	−4	0	52
		6		3.33	−16	−8	46
Planum polare (PP)^*^	**R**	**22**	**271**	**4.75**	**42**	**2**	**−20**
Temporal pole (TP)		38		3.79	54	8	−12
Temporal pole (TP)		38		3.75	34	16	−28
Seed in left postcentral gyrus [−44, 22, 62]							
**Planum polare (PP)**	**L**	**22**	**2,735**	**5.00**	**−48**	**−6**	**−2**
		22		4.87	−48	0	−14
Heschl’s gyrus (HG)		41/42		4.79	−52	−16	8
Planum temporale (PT)		42		4.72	−54	−30	14
**Planum polare/central operculum (PP/COp)**	**R**	**22/48**	**2,469**	**4.89**	**50**	**−2**	**0**
Central operculum (COp)		48		4.87	54	8	0
Planum polare (PP)		22		4.81	60	−2	2
Temporal pole (TP)		38		4.50	52	10	−12
Planum temporale (PT)		42		4.44	60	−22	12
Heschl’s gyrus (HG)		41/42		4.38	46	−16	8
**Postcentral gyrus (PoCG)**	**L**	**3/4**	**1,448**	**4.62**	**−36**	**−28**	**56**
Precentral/postcentral gyrus (PrCG/PoCG)		6/4		4.37	−44	−14	50
Postcentral gyrus (PoCG)		3/2		3.80	−34	−38	68
**Pallidum**	**L**	**—**	**446**	**4.21**	**−20**	**−8**	**−2**
Caudate		—		4.14	−18	0	20
Caudate		—		3.92	−20	10	20
Putamen		—		3.58	−22	−8	8
**Supplementary motor cortex (SMC)**	**L**	**6**	**427**	**4.39**	**−4**	**0**	**52**
		6		3.76	−4	2	68
**Caudate**	**R**	**—**	**365**	**3.87**	**20**	**−10**	**26**
		—		3.78	22	8	20
		—		3.34	16	0	26

*Note:* Peak voxels in clusters are shown in bold. Threshold: *P*-voxel < 0.001 and *P*-cluster < 0.05, FWE-corrected, if not marked otherwise. ^*^P-cluster = 0.055, FWE-corrected. *Abbreviations:* BA, Brodmann area; *k*, cluster size (number of voxels); Hem., hemisphere; L, left hemisphere; R, right hemisphere.

## Discussion

The goal of the current study was to investigate whether and how external sensory and internal cognitive factors modulate the balance of self-other integration and segregation during joint action. Therefore, we acquired MR-scans from pianists while they were performing duets with a partner. We manipulated the temporal compatibility of the partners’ action feedback (sensory) and their motor familiarity with each other’s part (cognitive). We expected cerebellar activity and behavioral segregation during subtle auditory asynchronies, neural indices of motor simulation and behavioral segregation during familiar conditions, and an interaction of both factors, potentially in the cerebellum. Our first main finding was stronger activity in fronto-parietal and cerebellar motor areas, increased audio-motor connectivity, as well as lower behavioral synchronization stability when pianists had practiced their partner’s part before. This, we will argue, shows that pianists internally simulated the partner’s part during the joint performance and tended towards stronger self-other segregation. Our second main finding was stronger cerebellar activity, weaker behavioral adaptation, and a correlation between these measures, when pianists’ actions were slightly out of sync following incongruent tempo instructions, but only when they were familiar with the partner’s part. As we will argue below, this may indicate that pianists used the motor simulation to predict their partner’s action timing, and shifted the balance towards self-other segregation when these simulation-based predictions were not precisely met. Taken together, our findings demonstrate how the interplay of sensory and cognitive parameters of a dyadic interaction can shift priorities between one’s own and the joint performance, regulated by fronto-temporo-parietal networks and the cerebellum.

### Motor simulation and auditory imagery of the partner’s part

The goal of our FAMILIARITY manipulation was to influence internal cognitive aspects of the joint performance, i.e. the availability of an internal model of the left-hand part performed by the partner, which was expected to shift the balance of self-other integration and segregation. Our results support this idea: Pianists showed (i) increased activity in premotor, parietal, and cerebellar sensorimotor regions, (ii) stronger audio-motor coupling, and (iii) lower behavioral synchronization stability when they were motorically familiar (as opposed to unfamiliar) with their partner’s part. Note that the motor output and auditory input was comparable between the 2 familiarity conditions: MR-pianists always played with the right hand (the melody) and heard both the melody and the bassline. The only difference was whether or not they had practiced the left-hand part prior to the experiment*.* We propose that the above-listed neural findings reflect the internal simulation and auditory imagery of the practiced left-hand part, including one’s own expressive timing and playing style, which has consequences for behavioral self-other integration and segregation as will be discussed in turn.

The increased fronto–parieto–cerebellar activity, including right (and less extensive left) premotor, primary somatosensory cortices (S1) and cerebellar lobules IV–V, during the performance of pieces with familiar left-hand parts is reminiscent of activations typically found during motor execution (Passingham 1997). However, given that motor execution was kept comparable between familiar and unfamiliar conditions, the effect is more likely to stem from additional processes related to the motor experience with the left-hand part performed by the partner. Indeed, previous studies reported activity in similar cortico-cerebellar areas during motor imagery, when participants mentally simulated the performance of actions without overt movement (Grèzes and Decety 2001; Hétu et al. 2013). Moreover, activation of these regions has been found during mere observation of other’s actions (Decety and Grèzes 1999; Errante and Fogassi 2020; Papitto et al. 2020) including visual or auditory perception of complex sequential actions such as dance or music performance that belonged to the observers’ motor repertoire (Calvo-Merino et al. 2005, 2006; Bangert et al. 2006; Lahav et al. 2007; Lee and Noppeney 2011; de Manzano et al. 2020). In light of these findings, it seems plausible to assume that the stronger sensorimotor activity during pieces with familiar left-hand parts reflects the motor simulation of these parts based on pianists’ motor knowledge and enhanced by the audio-input of that part performed by the partner. This interpretation is broadly consistent with the notion that bi-directional links between perception and action lead to increased resonance in the sensorimotor system when perceived actions closely match motor representations in an observer or co-actor (Schütz-Bosbach and Prinz 2007).

One may argue that the observed activity is not necessarily related to the interpersonal interaction between pianists, but may reflect processing differences between motor programs practiced with 2 hands (familiar) compared to only 1 hand (unfamiliar), which would also occur during solo performance. This may well be the case. However, previous studies have clearly demonstrated that a social context can influence motor simulation (Kokal et al. 2009; Novembre et al. 2012; Sacheli et al. 2018). For example, Novembre et al. (2012), who used a similar paradigm as our study, found enhanced motor activity for familiar left-hand parts only when pianists were duetting, not when they played their parts as solos. In the present data, brain activity during the second phrase may be informative, if taken as a proxy for solo performance, given that partners had no sensory contact during this time. Interestingly, a follow-up analysis showed no activation differences between familiar and unfamiliar left-hand parts in the second phrase. Although this finding should be interpreted cautiously (see section “Materials and methods”), it is in line with our interpretation that motor simulation is enhanced by a social (interaction) context, which should be further investigated by future studies.

Overall, this interpretation integrates well into theories that extend the concept of internal models from own actions to social interactions (e.g. Wolpert et al. 2003; Keller 2008; Keller et al. 2014, 2016; Pesquita et al. 2018; Müller et al. 2021). Originally, internal models have been described in the control of one’s own movements (Wolpert et al. 1995) where an internal simulation of that movement by a forward model (based on an efference copy of a motor command) helps to anticipate its sensorimotor consequences ahead of time and to smoothly adjust the movement if needed (Ramnani 2006; Kilteni et al. 2018; McNamee and Wolpert 2019). Later, the notion of internal models has been proposed to generalize to the anticipation of others’ actions allowing for seamless coordination between interaction partners (Wolpert et al. 2003; Lee and Noppeney 2011; Keller et al. 2014, 2016; Pesquita et al. 2018; Hadley and Pickering 2020; Müller et al. 2021), as supported by behavioral (Pezzulo et al. 2017; Sacheli et al. 2018) and EEG evidence (Kourtis et al. 2019). In line with this reasoning, our pianists may have generated sensory predictions of their partner’s ongoing performance by virtue of internal forward models of the familiar left-hand parts, while at the same time performing their own right-hand parts (Novembre et al. 2012, 2014; Bolt and Loehr 2021).

These simulation-based sensory predictions may be supported by the results of our psychophysiological interaction analysis that showed increased functional connectivity between (pre)motor areas, BG, and bilateral temporal regions during performance of familiar conditions, including Heschl’s gyrus (HG), planum temporale and polare (PT and PP), as well as the temporal pole (TP). The BG are known to support movement sequencing and rhythm processing (Grahn and Brett 2007; Teki et al. 2011b; Rajendran et al. 2018; Cannon and Patel 2021). The observed temporal areas are typically involved in auditory perception and musical imagery (Zatorre and Halpern 2005; Zhang et al. 2017; Martin et al. 2018), with TP being linked to higher order processes such as the recognition of familiar tunes (Hsieh et al. 2011), and the processing of musical melody and harmony (Brown et al. 2004), both functions that are plausibly relevant for sensory predictions of familiar partner actions. Overall, this audio-motor connectivity is in line with previous findings in single-participant studies, showing auditory-motor co-activations during both purely auditory (Haueisen and Knösche 2001; D’Ausilio et al. 2006; Grahn and Rowe 2009; Chen et al. 2012; Herholz et al. 2016; de Manzano et al. 2020) or purely motoric tasks (Bangert et al. 2001, 2006), as well as training-induced and performance-related increases of functional audio-motor connectivity (Segado et al. 2018; Wollman et al. 2018). The present data extend these findings from solo performance to joint action. Notably, both motor output and audio input were identical between familiar and unfamiliar conditions. This suggests that the enhanced audio-motor connectivity stems from the knowledge of the left-hand part and its auditory consequences, i.e. the action performed by the partner, rather than one’s own performance. This is in keeping with the idea that internal forward models are not only used for anticipating sensory consequences of self-performed actions, but also of familiar actions performed by others.

Finally, it seems important to say that internal forward models and the motor simulation of familiar actions include individual signatures of one’s own action strategy and style (Hilt et al. 2020). In piano performance, this pertains, amongst others, to one’s own expressive timing and playing style, depending on personal artistic choices as well as individual characteristics of each pianist’s neuromuscular system (Keller et al. 2007; Ragert et al. 2013; Van Vugt et al. 2013; Zamm et al. 2016). The individual temporal signature of the motor simulation may, hence, slightly differ from the temporal signature of the partner’s actual performance, which is likely to have consequences for self-other integration and segregation. Indeed, our behavioral data show lower synchronization stability (*SD* of asynchronies) between partners in pieces with familiar compared to unfamiliar left-hand parts (for similar results, see Ragert et al. 2013), although we did not observe differences in interpersonal coupling (cross-correlations at lag 0; see Novembre et al. 2016; Gugnowska et al. 2022). While differences in coupling may have been masked due to asymmetric adaptation strategies between pianists inside and outside the MR-scanner (see below), the differences in synchronization stability are in line with our hypotheses. They suggest a slightly more internal focus and shift towards stronger self-other segregation when pianists could rely on their own temporal signature of the left-hand part, and when they may have experienced subtle discrepancies between the motorically anticipated and the actually perceived timing of the partner’s action (Keller et al. 2007; Ginsborg and King 2012; Ragert et al. 2013; Novembre et al. 2014). In turn, without simulation-based sensory predictions of the partner’s timing in unfamiliar conditions, pianists may have adopted a stronger external focus and may have integrated other-produced sensory information more strongly into their own action plans, reflecting a shift towards stronger self-other integration.

Altogether, the neural and behavioral results jointly suggest that motor familiarity with a partner’s action can set a foundation for modulating the balance of self-other integration and segregation by virtue of internal forward models, including motor simulation and auditory anticipation in cortico-cerebellar audio-motor networks.

### Self-other segregation when simulation-based temporal predictions and partner’s action timing mismatch

To further assess how external sensory information, i.e. the temporal compatibility of both partners’ action feedback, influences the balance of self-other integration and segregation, we additionally manipulated the duos’ interpersonal synchrony to be relatively high or low. We did so by cueing pianists at the beginning of each trial to perform the second phrase at a predefined faster or slower tempo, either in congruent (e.g. both of them speeded up) or incongruent directions (e.g. one pianist speeded up, the other slowed down). It has been shown that anticipated tempo changes bias pianists’ performance tempo towards the impending, new tempo well before its execution (Repp 2001). Accordingly, in the case of incongruent tempo instructions, this induced subtle keystroke asynchronies between pianists already in the first phrase, despite the joint tempo of 120 bpm (see Fig. 2A), which replicates previous results (Novembre et al. 2016; Gugnowska et al. 2022). These asynchronies occurred irrespective of pianists’ (un)familiarity with the partner’s part and validate the experimental manipulation. As expected, they modulated the degree of interpersonal adaptation (Repp 2005; Repp and Su 2013), although only in MR-pianists who adapted less to their partners in incongruent than congruent trials (main effect of TEMPO in lag −1 cross-correlations; see end of the section “Discussion” for lag +1).

Interestingly, brain activity of the MR-pianists showed no overall differences between congruent and incongruent trials, although tempo congruency played a role in familiar conditions, as will be discussed below. This lack of a TEMPO main effect may be due to the subtlety and volatility of the asynchronies. Indeed, asynchronies were in the range of a few milliseconds and were shown to resolve after the first 3 keystrokes (Gugnowska et al. 2022; see also Fig. 1C), possibly too quickly to be captured with the low temporal resolution of fMRI.

Notably, however, both our behavioral and brain data suggest that these subtle interpersonal asynchronies were processed differently depending on whether pianists were familiar with their partner’s part (FAMILIARITY × TEMPO interactions): Only in familiar trials with incongruent (compared to congruent) tempo instructions, pianists showed (i) increased activity in sensorimotor lobule VIIIb of the left cerebellum, and (ii) decreased behavioral adaptation of the MR-pianists to their partners, reflected in lower lag −1 cross-correlations. Moreover, (iii) neural activity differences and differences in behavioral adaptation between congruent and incongruent familiar trials were negatively correlated. This indicates that the more the cerebellum was activated by temporal asynchronies, the less pianists adapted their performance to that of the partner. These results converge with those discussed above, suggesting a shift towards stronger self-other segregation when pianists experienced temporal discrepancies between the motorically anticipated (by virtue of internal forward models) and the actually perceived sensory feedback of their partner. The cerebellar activity may reflect both the mismatch detection between internal simulation-based feedback anticipations of familiar actions and external partner-produced sensory feedback, and/or the regulation of the behavioral adaptation. This is consistent with predictive coding approaches that posit that the brain attempts to minimize prediction errors both by updating predictions based on sensory input and by adapting behavior to generate predicted sensory effects (Vuust et al. 2022). Present results highlight the potential role of the cerebellum in these processes.

The cerebellum, including lobule VIIIb, has been frequently associated with internal forward models in the control of self-produced movements (Kawato and Gomi 1992; Wolpert et al. 1998; Bastian 2006; Ishikawa et al. 2016; Peterburs and Desmond 2016; Popa and Ebner 2019). This structure has been implicated in the detection of subtle perturbations of absolute (experience-based) time intervals (Teki et al. 2011b), motor timing in music performance and time perception more generally (Ivry et al. 2002; Grahn and Brett 2007; Zatorre et al. 2007; Chen et al. 2008a, 2008b). Most importantly, the cerebellum plays a crucial role in comparing motorically anticipated (i.e. simulation-based) and perceived sensory outcomes of own movements (Brown et al. 2006; Del Olmo et al. 2007; Chen et al. 2009; Sokolov et al. 2017), and in adapting behavior if necessary (Johnson et al. 2019). Recent evidence suggests that similar processes occur when self-produced movements are merely imagined and sensory feedback is produced externally (Kilteni et al. 2018). The present data further extend these findings to dyadic interactions, suggesting close links between the motor simulation of other-produced (familiar) movements and the auditory feedback produced by the partner (see also Sacheli et al. 2018). Overall, this interpretation is consistent with proposals that musicians not only run forward models of their own actions, but also of actions performed by their partners (Keller et al. 2014; Vesper et al. 2017; Bolt and Loehr 2021; Müller et al. 2021).

The cerebellar activity increase during familiar-incongruent trials was correlated with decreased behavioral adaptation of the MR-pianists to the performance timing of their partners (cross-correlation at lag −1). This behavioral pattern suggests a shift towards stronger self-other segregation and mirrors behavior when interacting with poorly- or overly-adaptive partners (Fairhurst et al. 2013), or when taking lead in joint performance (Fairhurst et al. 2014; Konvalinka et al. 2014; Heggli et al. 2019b; Heggli et al. 2021). It is conceivable that the subtle asynchronies were disturbing when they mismatched simulation-based feedback predictions (Repp and Keller 2010), making it computationally more efficient to suppress the partner-produced auditory feedback partially and to reduce behavioral adaptation to stabilize one’s own tempo (for similar modulations of feedback control in solo performance, see Pfordresher et al. 2014), and possibly to make one’s own behavior more predictable for the partner to follow (Vesper et al. 2011; Konvalinka et al. 2014; Novembre et al. 2019; Heggli et al. 2021). This idea is in line with the more general role of the cerebellum in modulating adaptive behavior following feedback-errors (Johnson et al. 2019) via inhibitory processes (Ishikawa et al. 2016; Peterburs and Desmond 2016). The previously reported functional connectivity between cerebellar lobule VIII and premotor/superior-parietal cortex, as well as the TPJ (Kipping et al. 2013) may further support the role of the cerebellum in integrating sensory and model-based action feedback, especially because the TPJ has been associated with modulations in self-other integration (Fairhurst et al. 2013; Heggli et al. 2021), e.g. depending on leader-follower roles (Vanzella et al. 2019). Altogether, the neural and behavioral findings highlight the important role of the cerebellum in linking cognitive and sensory factors of the interaction influencing the balance between self-other integration and segregation during joint music performance.

As a final note, pianists exhibited different adaptation strategies depending on whether they played inside the MR-scanner (lag −1) or outside (lag +1 cross-correlations). Pianists outside the scanner adapted overall more strongly to their partners than vice versa (main effect of LAG), and did so similarly across all conditions (no main effects or interaction for lag +1 cross-correlations). Previous studies associated such asymmetric adaptation patterns with different individual task constraints: For example, people with longer arms adapted more strongly to people with shorter arms than vice versa when carrying wooden planks together (Isenhower et al. 2010; see also: Vesper et al. 2013; Skewes et al. 2015; Era et al. 2018). The present data suggest that participants took the higher task demands of MR-pianists (playing in supine position) into account, and adapted more strongly and similarly across all conditions to compensate for performance fluctuations of their partner. This asymmetry may also explain why we did not find differences in interpersonal coupling strength (lag 0 cross-correlations) that have been typically reported by studies with carefully balanced leader-follower roles (Novembre et al. 2016; Gugnowska et al. 2022). Future studies may further explore the relationship between the neural activity of interactors performing under more similar conditions using dual-fMRI.

## Conclusion

The present study demonstrates that cortico-cerebellar audio-motor networks relay own internal motor knowledge and partner-produced sensory information during joint piano performance, and regulate the balance between self-other integration and segregation. The observed activity and functional connectivity changes for motorically familiar and temporally discrepant partner actions indicate that pianists covertly simulated the performance of their partner’s part and anticipated the respective auditory feedback by virtue of internal forward models of (familiar) partner actions. Discrepancies between these simulation-based feedback predictions and partner-produced sounds shifted the balance towards self-other segregation, orchestrated by the lateral cerebellum. These combined findings are in line with theories that extend the concept of internal models from self-produced actions to social interaction. Taken together, this study provides first insights into how different external sensory as well as internal cognitive factors influence the way musicians dynamically balance their resources to achieve synchrony during ensemble performance.

## Data and code availability statement

The consent form of the current study does not permit public archiving of the datasets analyzed during the experiment. Data can be made available upon request.
